# The impact of different suturing techniques for abdominal fascia closure on the Interleukin-6 expressions in *Rattus norvegicus*

**DOI:** 10.1186/s13104-020-05349-y

**Published:** 2020-10-30

**Authors:** Heryu Prima, Imam Sofii, Aditya Rifqi Fauzi, Ishandono Dachlan, Gunadi

**Affiliations:** 1 Digestive Surgery Division, Department of Surgery, Faculty of Medicine, Public Health and Nursing, Universitas Gadjah Mada/Dr. Sardjito Hospital, Yogyakarta, 55281 Indonesia; 2 Pediatric Surgery Division, Department of Surgery, Faculty of Medicine, Public Health and Nursing, Universitas Gadjah Mada/Dr. Sardjito Hospital, Yogyakarta, 55281 Indonesia; 3 Division of Plastic Reconstructive and Aesthetic Surgery, Department of Surgery, Dr. Sardjito Hospital, Faculty of Medicine, Public Health and Nursing, Universitas Gadjah Mada, Yogyakarta, Indonesia

**Keywords:** Abdominal fascial closure, Interleukin-6 expression, Incisional hernia, Large stitch, Small stitch

## Abstract

**Objective:**

Incisional hernia is a frequent complication of midline laparotomy. The suturing technique is an important determinant of the risk of developing an incisional hernia. Moreover, IL-6 has crucial roles in the wound-healing process. We aimed to compare the large stitch *vs*. small stitch technique for abdominal fascial closure on IL-6 expressions in rats.

**Results:**

Twenty rats were used. The small stitch group received small tissue bites of 5 mm and the large stitch group received large bites of 10 mm. The incisions of fascia were closed by running sutures. Animals were euthanized on days 4 and 7. Histological sections of the tissue-embedded sutures were analyzed for IL-6 expressions. Two-way ANOVA showed that rats in the small stitch group had similar IL-6 expressions on days 4 and 7 to those in the large stitch group (*p* = 0.36). In conclusion, the IL-6 expressions are similar between the small and the large stitch groups, implying that different suturing techniques might not have an impact on the incisional hernia occurrence.

## Introduction

Incisional hernia is a frequent complication of post-abdominal wall incisions and causes reoperation. In various meta-analysis and meta-regression studies, the mean incidence rate of incisional hernia at 23.8 months post-abdominal incision was 12.8% [1]. The occurrence of an incisional hernia has an important impact on the quality of life and body image of the patient [2]. The estimated cost of incisional hernia management reaches billions of US dollars annually [3]. Therefore, to reduce the incidence rate of incisional hernia requires optimal closure of the abdominal wall incision in order to save both cost and use of health facilities and reduce postoperative disability [1].

The median incision is the most commonly selected incision for open abdominal surgery [4]. A number of experimental and clinical studies have shown that the quality of stitching techniques is essential for the prevention of wound complications in the median incision [5]. Incisional hernia is the late complication of fascial disruption [6]. Fascia can more efficiently pick up and activate cellular elements that are important for the proliferative phase of the abdominal incision wound healing process. The proliferation phase of wound healing begins to dominate on the fourth day after the abdominal incision and fibroblasts are the most important cellular components during this phase [7]. Observation of fibroblast cellularity and collagen staining in rats showed increased levels on the seventh day after fascial incision [8]. Fibroblasts secrete pro-inflammatory cytokines, Interleukin-6 (IL-6), in response to strain [9]. IL-6 has a direct role in the proliferation and remodeling phase of wound healing by promoting collagen and angiogenesis deposition [10].

Many studies have shown a beneficial effect of the small stitch technique compared with large stitch in the prevention of incisional hernia. However, the studies did not focus on the effect of suturing techniques on IL-6 expression in the prevention of incisional hernia, so accordingly, this study aimed to compare the effects of the large stitch with the small stitch technique for abdominal fascial closure on IL-6 expressions in rats.

## Main text

### Material and methods

#### Animal subjects

An experimental study was conducted on Wistar rats (*Rattus norvegicus*). Twenty 170–200 g rats were selected for this study, which were adapted for a week prior to the research. Our study was in line with the guidelines of experimental animals: 3R (replacement, reduction, and refinement) and 5F (freedom of hunger and thirst, freedom from discomfort, freedom of pain, injury, or disease, freedom to fear and distress, and freedom to express natural behaviour). Therefore, we determined our subjects to be 20 rats. Subjects were divided into 4 groups, consisting of four interventional groups (each consisting of 5 rats): large stitch group and euthanized on day 4 (group 1), small stitch group and euthanized on day 4 (group 2), large stitch group and euthanized on day 7 (group 3), and small stitch group and euthanized on day 4 (group 4). All rats were euthanized by decapitation. All procedures were done under sedation using ketamine (Ikapharmindo, Jakarta, Indonesia) intramuscular injection 60 mg/kg after aseptic-antiseptic action with 1% povidone iodine. The anesthesia was maintained by additional doses as necessary. The animals were kept supine during the experiments. Incision in the abdominal skin of mice involved a length of 6 cm, then a 6-cm incision in the alba line. All stitches were placed at 5 mm from the edge of the wound for the small stitch group and at 10 mm from the edge of the wound to the large stitch group. Suture technique used continuous suture and suture material was polyvinylidene fluoride monofilament. Examination of IL-6 expression of paraffin blocks used immunohistochemical assay. Counting the number of IL-6 expressions used a counter in the Adobe Reader application. The expressions of IL-6 appeared as dark brown cells.

#### Statistical analysis

Data were subjected to statistical analysis using SPSS ver. 24 (IBM Corp., New York). Normality test was done and hypothetical tests comparing each interventional group preceded 2-way ANOVA. Significance was met if *p* < 0.05. The Medical and Health Research Ethics Committee of the Faculty of Medicine, Universitas Gadjah Mada approved the study (KE/FK/0151/EC/2018).

### Results

**Fig. 1:**
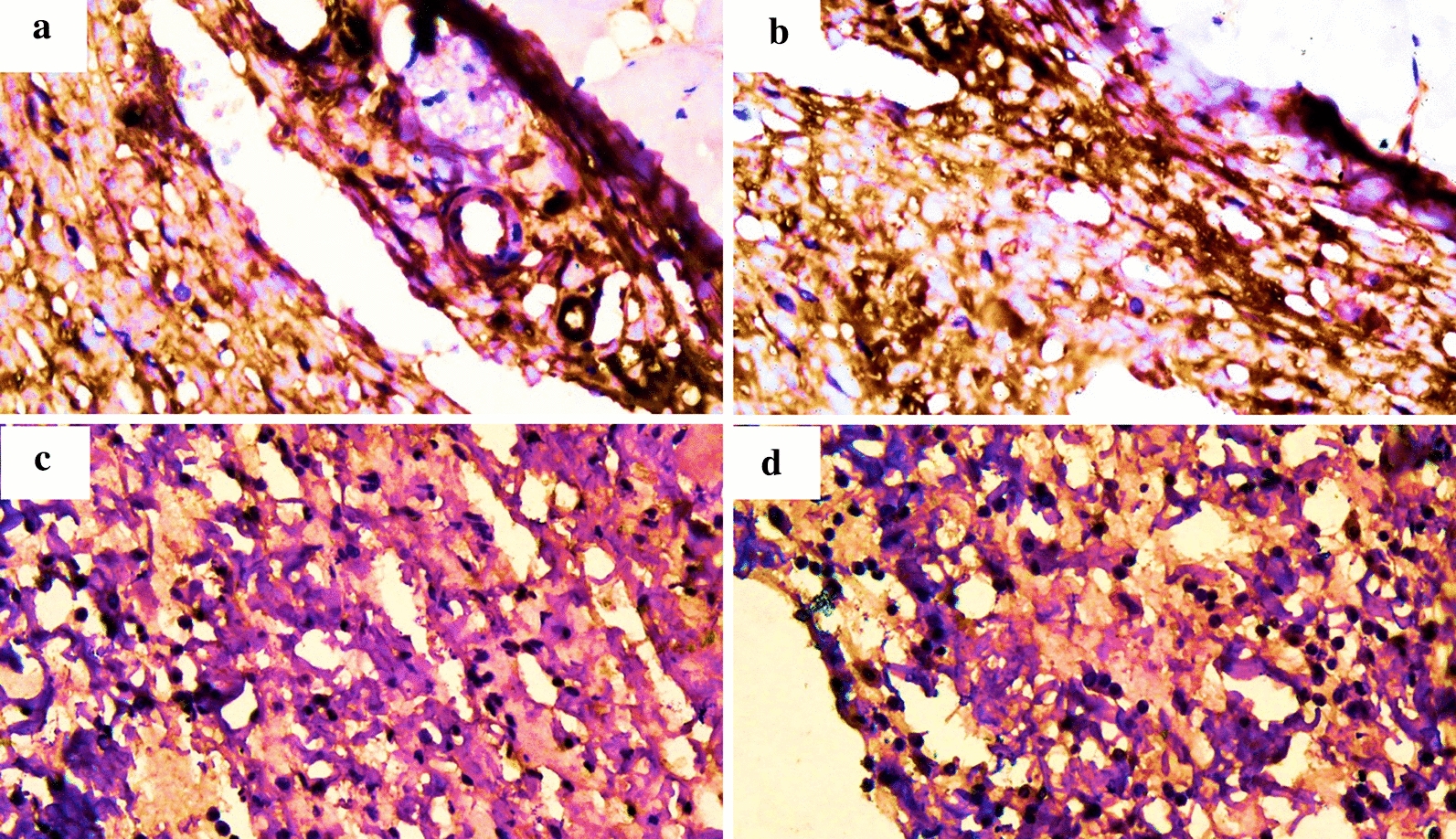
IL-6 expressions in the small stitch group on: **a** day 4 and **b** day 7, and the large stitch group on: **c** day 4 and **d** day 7. The IL-6 expressions were similar among groups and days (*p* > 0.05)

After immunohistochemical staining on tissue samples of each group, the expressions of IL-6 appeared as dark brown cells (Fig. 1).

Rats in the small stitch group had similar IL-6 expressions on days 4 and 7 to those in the large stitch group (*p* = 0.36) (Table 1).

**Table 1:** Comparison of IL-6 expressions between small and large stitch groups on days 4 and 7

	*IL-6* expressions		*p*-value
	Small stitch (mean ± SD)	Large stitch (mean ± SD)	
Day 4	38.4 ± 25.91	37.13 ± 22.75	0.36*
Day 7	69.67 ± 43.97	58.33 ± 35.02	

*IL-6* Interleukin-6, *SD* standard deviation; *p*-value was calculated using 2-way ANOVA

### Discussion

Here, we show that the effect of small and large stitch techniques is similar in the healing of abdominal fascial lesions as evidenced by similar IL-6 expression levels. IL-6 has an important role in the wound healing process by regulating the infiltration of leukocytes, angiogenesis, and collagen deposition. IL-6 has a direct role in the proliferation and remodeling of wound healing by promoting collagen and angiogenesis deposition [10].

Millbourn et al*.* [11] conducted a randomized trial study and concluded that short stitch was associated with low incidence of wound infection and incisional hernias. Multivariate analysis showed that the risk of infection was doubled and the incisional hernia risk was fourfold higher in the long stitch group. The small amount of IL-6 in the long stitch group on day 4 after incision may cause fascial wound healing disorders. Fascial wound healing disorders can be caused by failure in the acute phase of inflammation. IL-6 deficiency causes immune deficiency against infections and acute phase inflammatory disorders after tissue damage and infection [12].

Fortenyl et al*.* [13] compared the results of abdominal wall closure based on long stitch (10 mm) and short stitch (interval 5 mm) intervals using the same suture material which was extra-long term absorbable monofilament and concluded that small stitch interval with monofilament elastic suture is absorbed better than a large stitch interval in preventing the incisional hernia which is seen with ultrasound in 1 year after the median laparotomy. Experimental research conducted by Lin et al. [10] explains that in rats with IL-6 deficiency there is a decrease in the number of angiogenic and fibrogenic growth factors so that angiogenesis and collagen deposition are delayed. The study by Lee et al. [14] explains IL-6-deficient mice showed an early phase delay in wound healing, as well as in hyperglycemic mice. IL-6 was significantly higher (sixfold) in hyperglycemic animals at days 1, 7, and 10 [14]. Our study shows an almost significant change in IL-6 expression in the small stitch group between days 4 and 7. Collectively, angiogenesis and precipitation of collagen are indispensable for wound healing. It has been established that IL-6 deficiency causes angiogenesis and delayed collagen deposition [10].

## Conclusions

The expressions of IL-6 are similar between the small and the large stitch groups, implying that different suturing techniques might not have an impact on the incisional hernia occurrence.

## Limitations

Our study used a relatively small number of rats and only 1-week observation.

## Data Availability

All data generated or analyzed during this study are included in the submission. The raw data are available from the corresponding author on reasonable request.

## Abbreviations

IL-6: Interleukin-6
SD: Standard deviation
